# Quantitative Analysis of Spectinomycin and Lincomycin in Poultry Eggs by Accelerated Solvent Extraction Coupled with Gas Chromatography Tandem Mass Spectrometry

**DOI:** 10.3390/foods9050651

**Published:** 2020-05-18

**Authors:** Bo Wang, Yajuan Wang, Xing Xie, Zhixiang Diao, Kaizhou Xie, Genxi Zhang, Tao Zhang, Guojun Dai

**Affiliations:** 1 College of Veterinary Medicine, Yangzhou University, Yangzhou 225009, China; dz120180009@yzu.edu.cn; 2 Joint International Research Laboratory of Agriculture & Agri-Product Safety, Yangzhou University, Yangzhou 225009, China; yzwyj163@gmail.com (Y.W.); yzdzx163@gmail.com (Z.D.); gxzhang@yzu.edu.cn (G.Z.); zhangt@yzu.edu.cn (T.Z.); daigj@yzu.edu.cn (G.D.); 3 College of Animal Science and Technology, Yangzhou University, Yangzhou 225009, China; 4 Institute of Veterinary Medicine, Jiangsu Academy of Agricultural Sciences, Key Laboratory of Veterinary Biological Engineering and Technology, Ministry of Agriculture, Nanjing 210014, China; xiexing@jaas.ac.cn

**Keywords:** poultry eggs, spectinomycin, lincomycin, ASE, GC-EI/MS/MS

## Abstract

A method based on accelerated solvent extraction (ASE) coupled with gas chromatography tandem mass spectrometry (GC-MS/MS) was developed for the quantitative analysis of spectinomycin and lincomycin in poultry egg (whole egg, albumen and yolk) samples. In this work, the samples were extracted and purified using an ASE350 instrument and solid-phase extraction (SPE) cartridges, and the parameters of the ASE method were experimentally optimized. The appropriate SPE cartridges were selected, and the conditions for the derivatization reaction were optimized. After derivatization, the poultry egg (whole egg, albumen and yolk) samples were analyzed by GC-MS/MS. This study used blank poultry egg (whole egg, albumen and yolk) samples to evaluate the specificity, sensitivity, linearity, recovery and precision of the method. The linearity (5.6–2000 μg/kg for spectinomycin and 5.9–200 μg/kg for lincomycin), correlation coefficient (≥0.9991), recovery (80.0%–95.7%), precision (relative standard deviations, 1.0%–3.4%), limit of detection (2.3–4.3 μg/kg) and limit of quantification (5.6–9.5 μg/kg) of the method met the requirements for EU parameter verification. Compared with traditional liquid–liquid extraction methods, the proposed method is fast and consumes less reagents, and 24 samples can be processed at a time. Finally, the feasibility of the method was evaluated by testing real samples, and spectinomycin and lincomycin residues in poultry eggs were successfully detected.

## 1. Introduction

Spectinomycin and lincomycin are aminoglycoside and lincosamide antibiotics, respectively, and they have synergistic and complementary effects on each other’s antibacterial spectra and antibacterial mechanisms. Spectinomycin is an inhibitor of bacterial protein synthesis and acts on the 30S subunit of ribosomes, and its antibacterial mechanism mainly involves preventing the binding of messenger ribonucleic acid and ribosomes, thereby hindering the synthesis of proteins and resulting in bactericidal effects [1]. The antibacterial mechanism of lincomycin mainly consists of binding to the bacterial ribosomal 50S subunit, which inhibits peptide acyltransferase, hinders the synthesis of bacterial proteins and results in bactericidal effects [2]. Spectinomycin has strong antibacterial activity against Gram-negative bacteria and weak activity against Gram-positive bacteria, whereas lincomycin has no effect on Gram-negative bacteria but a strong antibacterial effect on Gram-positive bacteria. Therefore, spectinomycin and lincomycin are usually used in combination to treat infections with Gram-positive bacteria and Gram-negative bacteria and are widely used to treat piglet diarrhea and infection by *Mycoplasma hyopneumoniae* and *Mycoplasma pneumoniae*, which cause chronic respiratory diseases in chickens [3,4,5,6]. However, spectinomycin can damage the eighth cranial nerve, exert kidney toxicity and block neuromuscular transmission; lincomycin has strong side effects, damages the gastrointestinal tract and liver, and even causes anaphylactic shock and death. Thus, China and the European Union (EU) have listed spectinomycin as a banned drug for poultry eggs and set maximum residue limits (MRLs) of 300–5000 μg/kg for spectinomycin and 50–1500 μg/kg for lincomycin in animal-derived foods [7,8]. The MRLs in the United States are 100–4000 μg/kg for spectinomycin in chicken and cattle muscle and liver and 100–600 μg/kg for lincomycin in pig muscle and liver. In addition, the presence of the latter two drugs in other animal-derived foods has been banned [9]. Japan has set MRLs for spectinomycin of 500–5000 μg/kg and for lincomycin of 200–1500 μg/kg in animal-derived foods [10]. Thus, it is important to develop fast and efficient analytical methods to detect spectinomycin and lincomycin in poultry eggs.

To date, many methods have been used to measure spectinomycin and lincomycin in animal-derived foods and animal feedstuffs, including fluorescent latex immunoassay (FLI) [11], micellar electrokinetic capillary chromatography combined with ultraviolet detection (MEKC-UVD) [12], enzyme-linked immunosorbent assay (ELISA) [13], high-performance liquid chromatography with electrochemical detection (HPLC-ECD) [14,15], HPLC with fluorescence detection (FLD) [16], HPLC-UVD [17,18], HPLC with evaporative light-scattering detection (ELSD) [19,20], hydrophilic interaction chromatography with mass spectrometry (HILIC-MS) [21], HILIC tandem MS (MS/MS) [22], HPLC-MS [23], HPLC-MS/MS [24,25,26,27,28], gas chromatography–nitrogen phosphorus detection (GC-NPD) [29,30] and GC-MS [30]. The FLI, ELISA, ECD, FLD, UVD and ELSD methods have low sensitivity, specificity, recovery and precision and have many limitations. Molognoni et al. [22] developed a liquid–liquid extraction (LLE) method combined with HILIC-MS/MS for the determination of spectinomycin, halquinol, zilpaterol and melamine residues in animal feedstuffs with good recovery and precision. Juan et al. [27] reported an accelerated solvent extraction (ASE) method for the trace analysis of macrolide and lincosamide antibiotics in meat and milk using HPLC-MS/MS, and the method was fast, sensitive and automatic, making it suitable for the determination of macrolide and lincosamide residues in meat and milk. Tao et al. [30] established an ASE approach for extracting lincomycin and spectinomycin residues from swine and bovine tissues using GC-NPD and GC-MS. ASE is an automated extraction technology that is widely used for veterinary drug residue detection in animal food because of its advantages, such as rapid analysis, low organic solvent use and batch sample processing. Compared to liquid chromatography, gas chromatography has been reported less frequently for the detection of spectinomycin and lincomycin in animal foods. Moreover, the use of single-stage GC-MS has several difficult limitations, such as the inability to effectively exclude sample matrix-derived interferences, to confirm false positives and quasi-deterministic parameters and to quantify target compounds. However, a gas chromatography–tandem mass spectrometry (GC-MS/MS) method can effectively address these issues and accurately quantify target compounds. Thus, an ASE-GC-MS/MS method was developed to determine spectinomycin and lincomycin residues in poultry eggs. The method parameters were validated according to the EU [31] and the Food and Drug Administration (FDA) [32] validation requirements.

## 2. Materials and Methods

### 2.1. Chemicals and Reagents

Spectinomycin (97.9% standard) and lincomycin (98.9% standard) were purchased from the Food and Drug Control Agency (Beijing, China). *N*,O-bis(Trimethylsilyl)trifluoroacetamide (BSTFA, >99.0% standard) was obtained from Sigma-Aldrich (St. Louis, MO, USA). Sodium dodecyl sulfonate (SDS, ≥99.0% standard) was purchased from Sangon Biotech (Shanghai, China). Acetonitrile and methanol (HPLC grade) were acquired from Merck (Fairfield, OH, USA). Analytical-grade phosphoric acid (H_3_PO_4_), sodium hydroxide, acetic acid, n-hexane, potassium dihydrogen phosphate (KH_2_PO_4_) and trichloroacetic acid (TCA) were obtained from Sinopharm Chemical Reagent Co. (Shanghai, China). Ultrapure water was obtained from a PURELAB Option-Q synthesis system (ELGA Lab Waters, High Wycombe, Bucks, UK).

Standard stock solutions of spectinomycin and lincomycin at 1 mg/mL were prepared in pure methanol. The standard working solutions were obtained by diluting the standard stock solutions with pure methanol according to the test needs.

### 2.2. GC-MS/MS Analysis

The GC-MS/MS system consisted of a Trace 1300 gas chromatograph, a TSQ 8000 triple quadrupole tandem mass spectrometer and a Triplus RSH automatic sample injector, and the TraceFinder 3.0 software was used for the analysis (Thermo Fisher Corp., Waltham, MA, USA). GC separation was performed using the following temperature program: 160 °C for 1 min; a ramp at 25 °C/min to 250 °C, followed by a 1 min hold; and a ramp at 15 °C/min to 300 °C, followed by a 5 min hold. A Thermo Fisher TG-5MS amine column (30 m × 0.25 mm; inside diameter (i.d.), 0.25 µm) was used. The GC was operated in splitless mode with a carrier gas (helium, 99.999% standard, 60 psi) flow rate of 1.0 mL/min. The injector temperature was held at 280 °C, and the injection volume was 1.0 µL.

The MS/MS system was equipped with an electron impact (EI) source and used in full scan mode and selected reaction monitoring (SRM) mode. The typical MS parameters were as follows: ionization voltage, 70 eV; ion source temperature, 280 °C; and transfer line temperature, 280 °C. The retention times and relevant MS parameters are presented in Table 1.

### 2.3. Preparation of the Samples

Considering that consumers have separate uses for whole eggs, albumens and yolks in hen, duck and goose eggs, we studied the elimination of spectinomycin and lincomycin residues in whole eggs, albumens and yolks. Because pigeon and quail eggs are relatively small, consumers generally use these as whole eggs. Thus, blank hen, duck and goose eggs were collected as whole eggs, albumen and yolk samples, and blank pigeon and quail eggs were collected as whole egg samples. Blank hen, duck and goose eggs (whole eggs, albumens and yolks) as well as pigeon and quail eggs (whole eggs) were separately homogenized, divided and frozen. In this work, LLE and ASE were used to extract the poultry egg samples, which were then cleaned up by SPE and finally derivatized.

#### 2.3.1. Liquid–Liquid Extraction

Homogenized poultry eggs (2.0 ± 0.02 g) were precisely weighed and then added to 10 mL of 0.01 M KH_2_PO_4_ solution (pH 4.0). The sample was vortexed for 5 min at 2000× *g*, homogenized ultrasonically for 10 min and then centrifuged for 10 min at 8000× *g*. The extraction solution was collected, and the sample was extracted again. The two extracts were combined, and 5 mL of n-hexane was added. The mixture was vortexed for 5 min at 2000× *g* and then centrifuged for 10 min at 8000× *g*. After degreasing twice with n-hexane, the extract was added to 5 mL of 3% TCA solution, vortexed for 5 min at 2000× *g* and then centrifuged for 10 min at 8000× *g*. The liquid–liquid extraction procedure was performed according to the National Food Safety Standard (GB 29685-2013) [33].

#### 2.3.2. Accelerated Solvent Extraction

Homogenized poultry eggs (2.0 ± 0.02 g) and 4.0 g of diatomaceous earth were fully ground, and then sample preparation was performed. The fat-removal and extraction parameters for the ASE350 instrument (Thermo Fisher Scientific Co. Ltd., Waltham, MA, USA) were as follows: 60 °C, 1500 psi, a static extraction time of 5 min and a nitrogen purge time of 60 s. One extraction was performed with a total solvent rinse of 40% and n-hexane to remove the fat, and two extractions were then performed with a total solvent rinse of 50% and 0.01 M KH_2_PO_4_ solution (pH 4.0) to extract the analytes, after which the sample extract was collected.

#### 2.3.3. Solid-Phase Extraction

After the sample was processed by LLE or ASE, 10% NaOH solution was added to the extract to adjust the pH to 5.8 ± 0.2, 2 mL of 0.2 M SDS solution was added, and the sample was then vortexed for 1 min. After standing for 15 min, the extract was cleaned up by SPE with an Oasis PRiME HLB cartridge (3 mL/60 mg, Waters Corp., Milford, MA, USA) that had been activated and equilibrated by the addition of 3 mL of methanol, 3 mL of ultra-pure water and 3 mL of 0.02 M SDS solution. After 20 mL of the extracts was added to the Oasis PRiME HLB cartridge at a constant rate (2.0 mL/min) and allowed to completely pass through the cartridge, 9 mL of ultrapure water was added in three portions for rinsing. Finally, 6 mL of methanol was used to elute the two target compounds.

### 2.4. Derivatization Reaction

After the extract was dried under a stream of nitrogen at 40 °C, 200 µL of BSTFA and 100 µL of acetonitrile were sequentially added to the sample, which was then vortexed for 1 min. Then, the mixture was placed in a 75 °C oven for 60 min. After the derivatization reaction was complete, the mixture was cooled to room temperature and dried under a stream of nitrogen at 40 °C. Finally, 2 mL of n-hexane was added to the sample to dissolve the residue, and the resulting solution was vortexed for 1 min and passed through a 0.22 μm organic phase needle filter into the GC-MS/MS system.

### 2.5. Quality Parameters

Seven spiked concentration levels for the two analytes were used to establish the linear regression equations: the limit of quantification (LOQ) and 50, 100, 500, 1000, 1500 and 2000 μg/kg for spectinomycin and the LOQ and 10, 20, 50, 100, 150 and 200 μg/kg for lincomycin. The peak areas as a function of the analyte concentration were used to establish standard working curves. The correlation coefficients (*R*^2^ values) were determined and should all have been ≥0.9991. The other parameters were tested according to the EU [31] and the FDA requirements [32], and the TraceFinder 3.0 software (Thermo Fisher Corp., Waltham, MA, USA) was used for the analysis.

## 3. Results and Discussion

### 3.1. Optimization of the ASE Conditions

Due to the complexity of the matrices of animal-derived foods, the detection of veterinary drug residues in such foods usually requires sample pretreatment involving extraction and clean-up to avoid clogging the chromatography column and contaminating the instrument. Several methods, such as LLE [22,24,26], solid-phase extraction (SPE) [16,21], core-shell molecularly imprinted solid-phase extraction (CSMISPE) [18] and ASE [27,30], have been developed for the extraction of spectinomycin and lincomycin from animal tissues, meat, milk and animal feedstuffs as well as from swine, calf and chicken plasma. Compared with the LLE, SPE and CSMISPE methods, the ASE method has the advantages of a short extraction time, lower consumption of organic reagents and batch sample processing. Therefore, in this study, the ASE method was used to extract spectinomycin and lincomycin from poultry eggs, and the analyte recoveries were compared with those for the LLE method.

**Figure 1:**
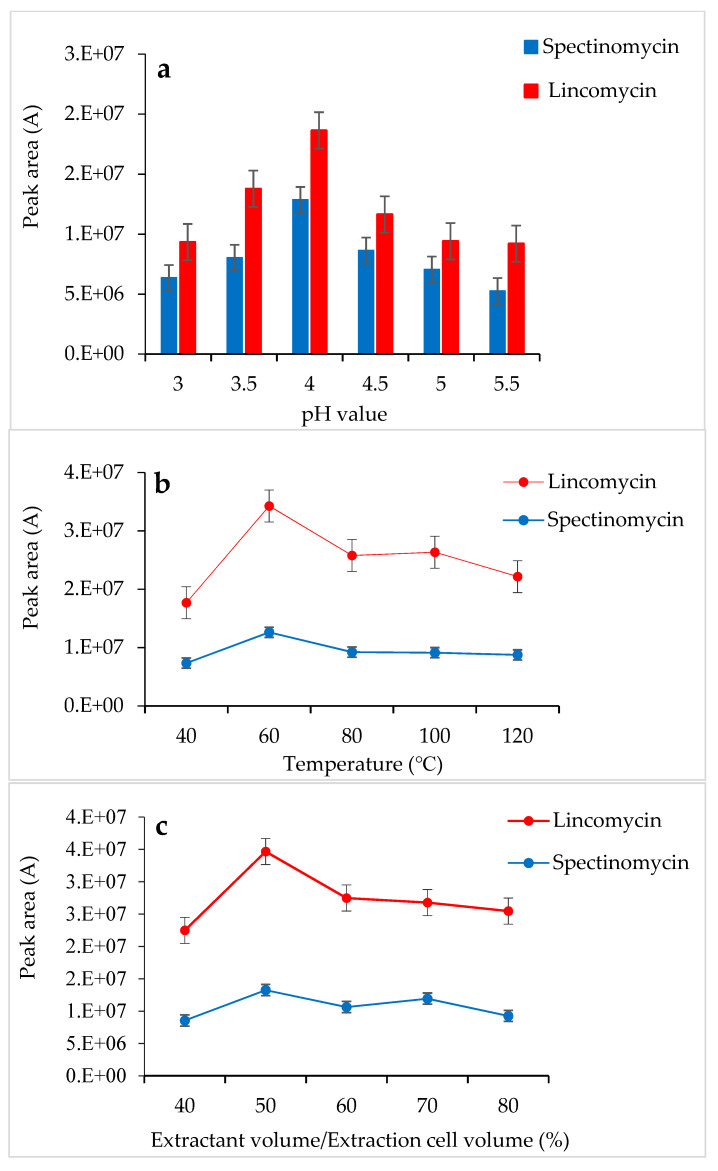
Effects of pH (**a**), temperature (**b**) and extractant volume (**c**) on the extraction efficiency of accelerated solvent extraction (ASE).

Tao et al. [30] used a 0.01 M KH_2_PO_4_ solution as an extractant to successfully extract spectinomycin and lincomycin from animal tissues. Based on the chemical properties of spectinomycin and lincomycin, a 0.01 M KH_2_PO_4_ solution was also selected as the extractant in the present study. In this experiment, the pH of the 0.01 M KH_2_PO_4_ solution was adjusted with H_3_PO_4_, and the effects of different pH values (3.0–5.5) on the response values of the two compounds were compared. When the 0.01 M KH_2_PO_4_ solution (pH 4.0) was used as the extractant, the response values of spectinomycin and lincomycin were the highest (Figure 1a). Thus, the 0.01 M KH_2_PO_4_ solution (pH 4.0) was finally selected as the extractant in this study. At 1500 psi, the effects of the temperature (40 °C, 60 °C, 80 °C, 100 °C and 120 °C), the amount of extractant (40%, 50%, 60%, 70% and 80% of the extraction cell volume), and the number of extractions (1 and 2 static cycles) on the recovery of spectinomycin and lincomycin from poultry eggs were compared. Firstly, using the 0.01 M KH_2_PO_4_ solution (pH 4.0) as the extractant and the optimal conditions for ASE extraction temperature were tested under 1500 psi, and the optimal extraction temperature was determined to be 60 °C (Figure 1b). Secondly, under the conditions of 1500 psi, 60 °C and using the 0.01 M KH_2_PO_4_ solution (pH 4.0) as the extractant, we optimized the amount of extractant and the number of extractions, and a 50% extraction cell volume and two static cycles obtained the best response value (Figure 1c). Thus, the optimal extraction conditions for the ASE method were as follows (Figure 1): 60 °C, 1500 psi, a 0.01 M KH_2_PO_4_ solution (pH 4.0) as the extractant, 50% extraction cell volume, static extraction for 5 min, one degreasing cycle and two static cycles.

### 3.2. Optimization of the SPE Conditions

**Figure 2:**
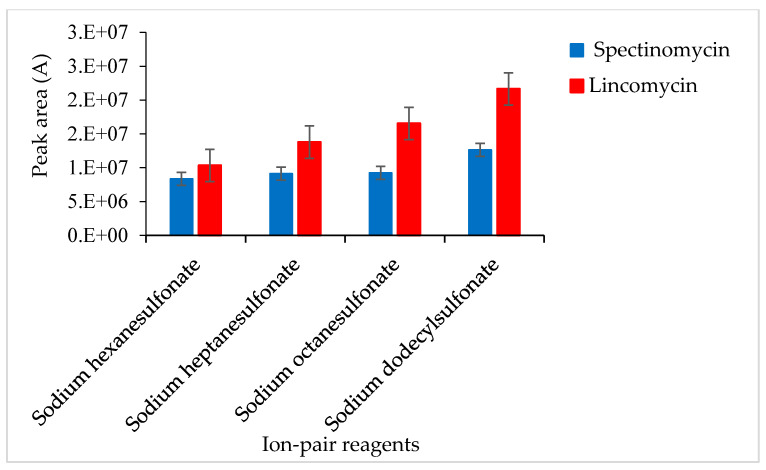
Effects of different ion-pair reagents on the recovery of spectinomycin and lincomycin.

An ion-pair reagent can be combined with the analyte to form an ion-pair and become neutral so that the analyte molecules are retained on the chromatographic column. A test revealed that the ion-pair reagent was susceptible to pH-induced changes: slight changes in pH affected the ion-pair reagent and, consequently, the recoveries of the target compounds. To solve this problem, after LLE or ASE, 10% NaOH was added to the sample extract to adjust the pH (to 5.4, 5.6, 5.8, 6.0 and 6.2), and then 2 mL of 0.2 M SDS solution was added to change the polarity. Adjusting the pH of the extract to 5.8 ± 0.2, the response value of the target was slightly improved. SPE cartridges were used to isolate spectinomycin and lincomycin from poultry eggs. The effects of different ion-pair reagents (sodium hexane sulfonate, sodium heptane sulfonate, sodium octane sulfonate and sodium dodecyl sulfonate) on the recoveries of the target compounds were compared. Sodium dodecyl sulfonate yielded the highest responses for the quantitative ion pairs (spectinomycin: *m*/*z* 201.1 > 75.0, lincomycin: *m*/*z* 126.1 > 42.0), which resulted in higher analyte recovery (Figure 2). Therefore, a 0.02 M sodium dodecyl sulfonate solution was used to equilibrate the SPE cartridge. This study compared C_18_ cartridges (6 mL/500 mg, Agela Technologies, Tianjin, China), PCX cartridges (6 mL/500 mg, Agela Technologies), and Oasis PRiME HLB cartridges (3 mL/60 mg, Waters Corp) in terms of the target compound recoveries. The C_18_ cartridge (6 mL/500 mg) produced interferences and did not effectively clean up the samples. The PCX cartridge (6 mL/500 mg) resulted in poor peak shapes and recoveries of less than 70%. The Oasis PRiME HLB cartridge (3 mL/60 mg) effectively cleaned up the samples and yielded recoveries above 80%. The Oasis PRiME HLB cartridge is a new type of solid-phase extraction cartridge that can remove 99% of the phospholipid matrix interferences in the sample, which minimizes the matrix effect of mass spectrometry, resulting in more stable data, a longer column life cycle, less instrument maintenance and less downtime. Therefore, the Oasis PRiME HLB cartridge (3 mL/60 mg) was used for sample clean-up.

After the optimization of the extraction and clean-up conditions, the effects of the LLE-SPE and ASE-SPE methods on the recoveries of spectinomycin and lincomycin from poultry eggs were compared. The results (Table 2) show that the recoveries for the ASE-SPE method were higher than those for the LLE-SPE method. Therefore, the ASE-SPE method was used to extract and clean up spectinomycin and lincomycin residues in poultry eggs.

### 3.3. Optimization of the GC-MS/MS Analysis

**Figure 3:**
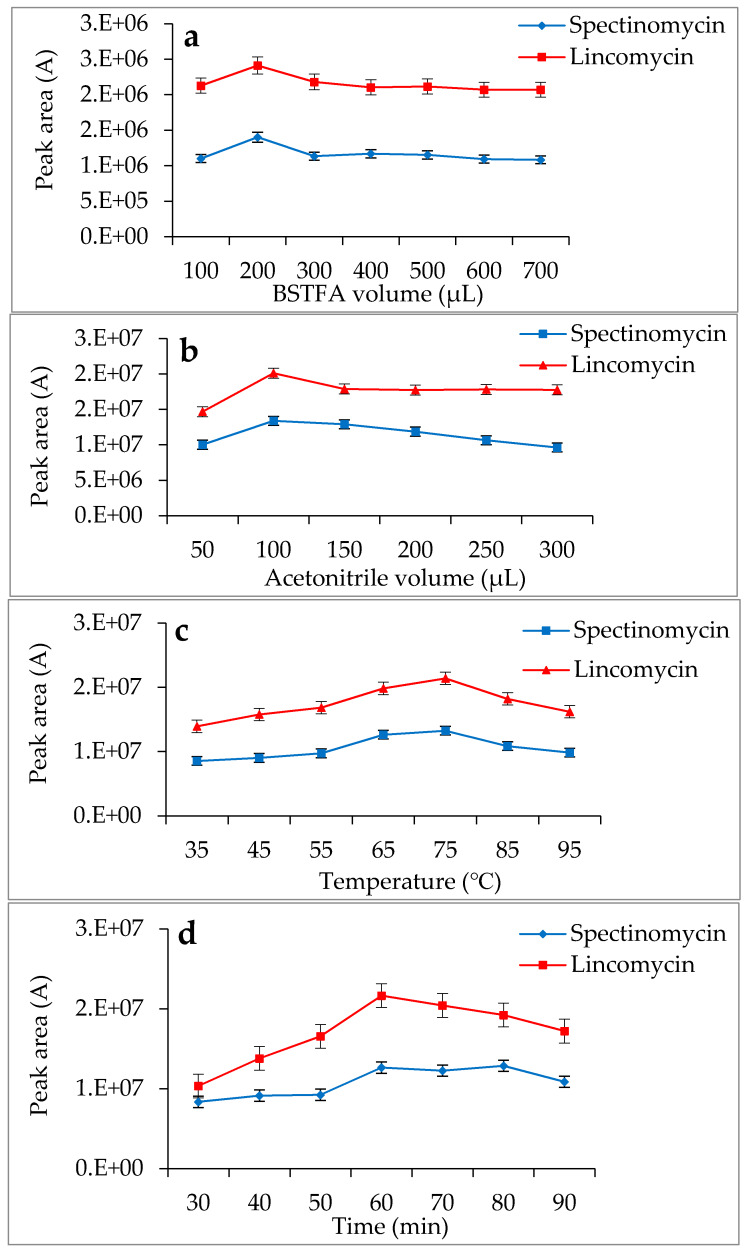
Effects of *N*,O-bis(Trimethylsilyl)trifluoroacetamide (BSTFA) volume (**a**), acetonitrile volume (**b**), temperature (**c**) and time (**d**) on the derivatization reaction.

**Figure 4:**
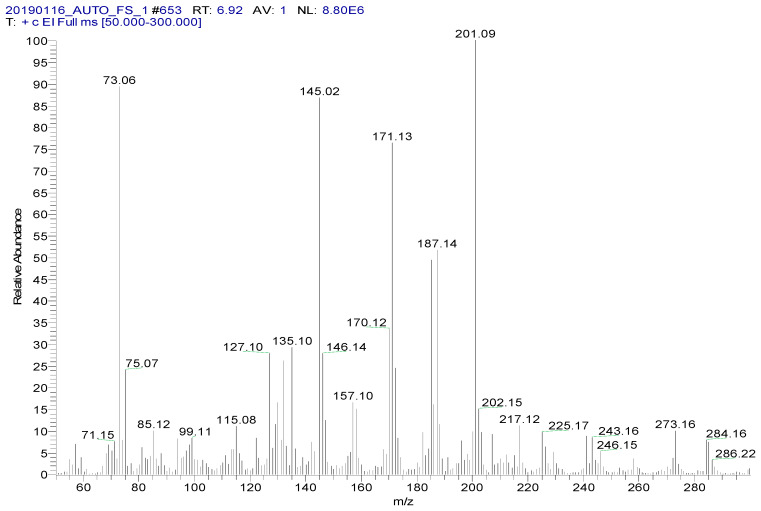
MS spectrum of spectinomycin-TMS.

**Figure 5:**
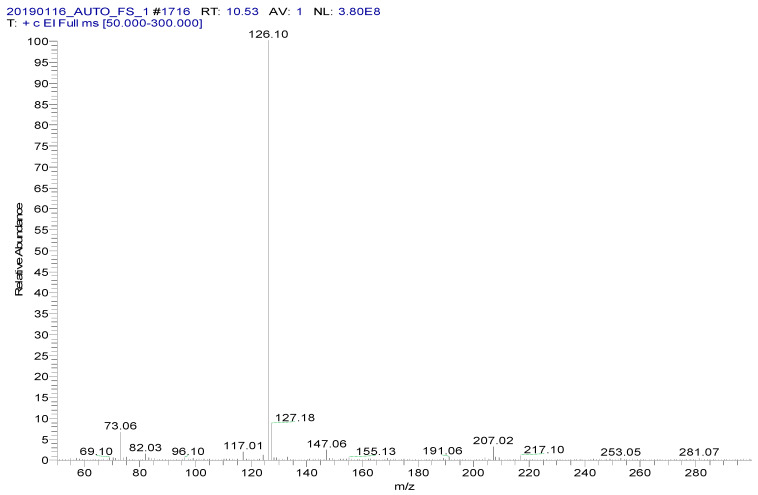
MS spectrum of lincomycin-TMS.

Spectinomycin and lincomycin are highly polar compounds and cannot be detected directly by GC techniques. Usually, derivatization is required to reduce the polarity and boiling point of these compounds before GC detection. Tao et al. [30] reported the successful detection of spectinomycin and lincomycin in animal tissues by a GC method after derivatization by BSTFA. Thus, BSTFA was used as the derivatization reagent in the present work, and the above method of optimizing the ASE parameters was used to optimize the following derivatization conditions: the amount of BSTFA (100–700 μL), amount of acetonitrile (50–300 μL), temperature (35–95 °C) and time (30–90 min). The optimal derivatization conditions (Figure 3) were 75 °C, 60 min, 200 µL of BSTFA and 100 µL of acetonitrile, under which spectinomycin and lincomycin were derivatized to spectinomycin- trimethylsilyl (TMS) and lincomycin-TMS (Figure 4 and Figure 5). After derivatization, BSTFA was removed by drying the sample under a stream of nitrogen. Excess BSTFA crystallizes easily and will plug and damage the column. TMS-derivatized products are easily hydrolyzed and stable for 24 h. Therefore, TMS-derivatized products should be analyzed by GC-MS/MS within 24 h.

**Figure 6:**
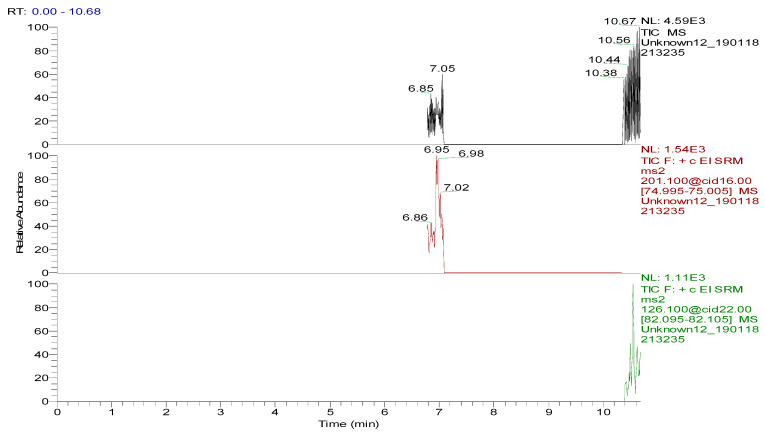
Total ion chromatogram and extracted ion chromatograms of a blank hen whole egg sample.

**Figure 7:**
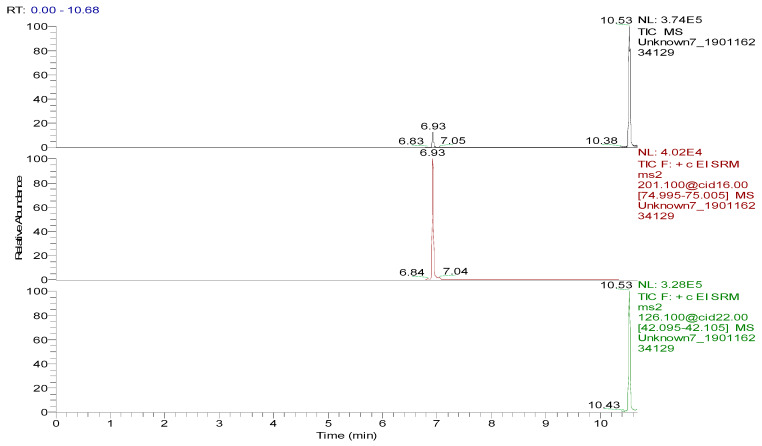
Total ion chromatogram and extracted ion chromatograms of a blank hen whole egg sample spiked with 50.0 µg/kg spectinomycin (retention time (RT), 6.93 min) and 50.0 µg/kg lincomycin (RT, 10.53 min).

Several capillary columns, including DB-1 (30 m × 0.25 mm i.d., 0.25 µm), HP-5 (30 m × 0.25 mm i.d., 0.25 µm) and Rtx-5 (30 m × 0.25 mm i.d., 0.25 µm), have been reported for the detection of spectinomycin and lincomycin in animal-derived foods and were tested herein. According to previous reports [29,30], lincomycin and spectinomycin derivatives have moderate polarities and low boiling points, so nonpolar and moderately polar capillary columns are usually used to detect these two compounds. The inner surface of the moderately polar TG-5MS (30 m × 0.25 mm i.d., 0.25 µm) capillary column has been chemically treated to reduce the tailing of active basic compounds and increase the detection of amines. Therefore, a TG-5MS (30 m × 0.25 mm i.d., 0.25 µm) capillary column was selected to analyze spectinomycin and lincomycin residues in poultry eggs. Next, the oven temperature program was optimized to decrease the retention time (RT) of the target compounds (spectinomycin and lincomycin, 6.93 and 10.53 min) and shorten the total run time. Analysis was performed in full scan mode and SRM mode to identify precursor and product ions. In this study, two monitored ion pairs were selected for the qualitative and quantitative analysis of the target compounds. The derivatized products were analyzed under the optimized GC-MS/MS conditions. The total ion chromatogram (TIC) and extracted ion chromatograms (XICs) of a blank hen whole egg sample are shown in Figure 6. The TIC and XICs of the quantitative ions from the blank hen whole egg spiked with 50.0 µg/kg spectinomycin and 50.0 µg/kg lincomycin (Figure 7) showed that spectinomycin and lincomycin in hen whole eggs could be effectively separated with sharp peaks and no tailing.

### 3.4. Bioanalytical Method Validation

The specificity of the method for analyzing blank poultry eggs was determined by comparing Figure 6 and Figure 7. Figure 6 shows that the blank hen whole egg sample did not contain spectinomycin and lincomycin. The blank poultry egg samples were extracted and cleaned up by the ASE-SPE method to obtain a blank matrix extract. The standard working solutions of spectinomycin and lincomycin and the reagents required for the abovementioned derivatization reaction were sequentially added to the blank matrix extract for derivatization. The standard curve was constructed from the GC-MS/MS analysis of the samples at the seven concentration levels. The linear ranges of spectinomycin and lincomycin were LOQ–2000 μg/kg and LOQ–200 μg/kg, respectively. The regression equation and determination coefficient data are listed in Table 3. According to the EU guidelines [31], the recovery and precision (intraday precision and interday precision) of the developed GC-MS/MS method were validated at the LOQ and at 0.5, 1.0 and 2 MRL (*n* = 6 at each level) for each drug in the poultry egg samples. In particular, 4000 μg/kg spectinomycin was added to the blank poultry egg sample; after extraction and purification by the ASE-SPE method, the sample was diluted with blank matrix extract 2-fold before the derivatization reaction was performed to ensure that the detected concentration of the sample was in the linear range. The measured concentration was multiplied by 2 to obtain the actual concentration of the original sample. By this method, the recovery and precision of measuring 4000 μg/kg spectinomycin in poultry eggs were evaluated. As shown in Table 4 and Table 5, the recoveries of spectinomycin and lincomycin in the blank hen, duck and goose egg (whole egg, albumen and yolk) samples as well as in the pigeon and quail egg (whole egg) samples were 80.0%–95.7%, and the relative standard deviations (RSDs) were 1.0%–3.4%. In addition, the intraday RSDs were 1.9%–6.0%, and the interday RSDs were 2.2%–6.7%. These data indicate that the recovery and precision of the method meet the EU [31] and FDA [32] requirements for methodological parameters.

Blank matrix extracts of the hen, duck and goose egg (whole egg, albumen and yolk) samples as well as of the pigeon and quail egg (whole egg) samples were prepared, and the spectinomycin and lincomycin standard working solutions were added to the blank matrix extract, derivatized and detected by GC-MS/MS. The concentrations corresponding to signal-to-noise (S/N) ratios of 3 and 10 for the target compounds were set as the limit of detection (LOD) and LOQ, respectively, of the target compounds in the hen, duck and goose egg (whole egg, albumen and yolk) samples as well as in the pigeon and quail egg (whole egg) samples. As shown in Table 3, the LODs of spectinomycin and lincomycin in the hen, duck, goose, pigeon and quail egg (whole egg) samples were 3.1, 3.5, 3.5, 4.0 and 3.8 μg/kg and 3.1, 2.8, 3.5, 3.9 and 4.3 μg/kg, respectively, and the LOQs of spectinomycin and lincomycin in the same poultry egg samples were 6.0, 6.3, 7.1, 8.0 and 7.6 μg/kg and 8.4, 6.5, 8.5, 9.5 and 8.2 μg/kg, respectively. The results for the LOD and LOQ of spectinomycin and lincomycin in the hen, duck and goose egg (albumen and yolk) samples are shown in Table 3. These LOQs and LOQs are relatively low, and the method is therefore highly sensitive and accurate.

### 3.5. Comparison of Different Detection Methods

Various analytical methods, including HPLC-FLD [16], HPLC-UVD [18], HILIC-MS/MS [22], HPLC-MS [23], HPLC-MS/MS [24,27], GC-NPD [30] and GC-MS [30], have been used to detect spectinomycin and lincomycin in meat, milk, feedstuffs, honey and animal tissues as well as in swine, calf and chicken plasma. Negarian et al. [18] established an HPLC-UVD method that showed better recovery (80.0%–89.0%) and precision (3.0%–3.9%) for the detection of lincomycin in milk and used CSMISPE to extract and clean up milk samples. Sin et al. [24] developed an LLE method to extract lincomycin from animal tissues and bovine milk. The average recoveries of lincomycin from animal tissues and bovine milk samples were 93.9%–107%, with a precision of 1.3%–7.8%. The LODs and LOQs of this method were 1.5–8.8 μg/kg and 25.0–50.0 μg/kg, respectively. Juan et al. [27] reported an ASE-HPLC-MS/MS method for the simultaneous determination of macrolide and lincosamide antibiotics in meat and milk. ASE is an automated technology that uses solvents at a relatively high pressure and a temperature below the critical points. Compared with LLE and SPE, ASE improves the work efficiency and reduces the amount of extractant required for analysis. Tao et al. [30] established GC-NPD and GC-MS methods for the determination of spectinomycin and lincomycin residues in animal tissues. Animal tissue samples were extracted by ASE, cleaned up with SPE cartridges and detected by GC-NPD and GC-MS. The average recoveries with the GC-NPD and GC-MS methods were 73.0%–97.0% and 70.0%–93.0%, and the RSDs were less than 17% and 21%, respectively. We compared the analysis time, sensitivity and recovery for spectinomycin and lincomycin analysis using different extraction and detection methods. As shown in Table 6, HPLC or GC with MS or MS/MS detection yielded higher sensitivity and precision than FLD, UVD and NPD.

ASE is an automated extraction technology that effectively improves the work efficiency, and 24 samples can be processed simultaneously in the same batch. In this study, LLE and ASE were used to effectively extract poultry egg samples. However, the LLE method is complicated and time- and reagent-consuming. After comparing sample pretreatment methods, we selected ASE for the extraction of spectinomycin and lincomycin residues from poultry eggs. Moreover, GC-MS/MS has higher sensitivity and precision than GC-MS. In this study, the parameters of ASE and GC-MS/MS were optimized to successfully detect spectinomycin and lincomycin in poultry eggs. The newly developed ASE-GC-MS/MS method provides new techniques and a scientific basis for the detection of spectinomycin and lincomycin residues in poultry eggs.

### 3.6. Real Sample Analysis

To evaluate the feasibility and accuracy of the newly developed method, we analyzed real samples using ASE-GC-MS/MS. One hundred and fifty commercial poultry eggs (30 hen eggs, 30 duck eggs, 30 goose eggs, 30 pigeon eggs and 30 quail eggs) were purchased from a local supermarket. Each poultry egg sample was processed in accordance with the sample pretreatment method described above and labeled, and each sample was detected and analyzed by the GC-MS/MS method. The target compounds were not detected in duck, goose, pigeon and quail eggs; only hen eggs were found to contain lincomycin residues (11.5 μg/kg less than the MRL). Therefore, the developed ASE-GC-MS/MS method can be applied to quantify spectinomycin and lincomycin in poultry egg samples.

## 4. Conclusions

In this study, we successfully developed a rapid, sensitive and specific ASE-GC-MS/MS method for the determination of spectinomycin and lincomycin residues in poultry egg samples. ASE is a promising technique for the preparation of animal-derived food samples. The developed method is accurate, has high recovery and precision, and fulfills the validation requirements of the Ministry of Agriculture of the People’s Republic of China, the EU and the FDA. The analysis of real samples showed that this new method is feasible and can detect spectinomycin and lincomycin residues in poultry egg samples.

## Figures and Tables

**Table 1:** Retention times and relevant mass spectrometry (MS) parameters for the analytes.

Analyte	Retention Time (min)	Molecular Weight (*m*/*z*)	Mass Transitions (*m*/*z*)	Collision Energy (eV)
Spectinomycin	6.93	332.15	201.1 > 75.0 * 201.1 > 185.1	16 8
Lincomycin	10.53	406. 21	126.1 > 42.0 * 126.1 > 82.0	22 22

Note: * Ion pair used for quantification.

**Table 2:** Comparison of the effects of the extraction method on the recoveries of 50 μg/kg spectinomycin and lincomycin in poultry eggs (%) (*n* = 6). Liquid–liquid extraction, LLE; solid-phase extraction, SPE.

Analyte	Matrix	Sample Preparation Method	
		LLE-SPE	ASE-SPE
Spectinomycin	Hen whole egg	80.5 ± 1.6	86.0 ± 1.5
	Albumen	81.4 ± 1.4	86.6 ± 2.0
	Yolk	81.2 ± 1.7	86.6 ± 2.1
	Duck whole egg	80.9 ± 3.4	86.1 ± 2.7
	Albumen	74.4 ± 1.4	85.7 ± 2.2
	Yolk	82.1 ± 3.4	87.1 ± 1.3
	Goose whole egg	79.2 ± 2.2	87.7 ± 1.7
	Albumen	81.1 ± 1.6	83.4 ± 1.4
	Yolk	80.9 ± 3.7	85.9 ± 1.2
	Pigeon whole egg	80.5 ± 2.7	87.7 ± 2.2
	Quail whole egg	79.0 ± 3.2	87.2 ± 1.9
Lincomycin	Hen whole egg	79.4 ± 1.6	84.8 ± 1.6
	Albumen	78.7 ± 2.7	85.8 ± 2.2
	Yolk	81.3 ± 3.3	84.8 ± 2.0
	Duck whole egg	75.4 ± 1.7	85.4 ± 2.6
	Albumen	76.0 ± 2.4	93.4 ± 2.3
	Yolk	83.5 ± 1.6	87.9 ± 2.1
	Goose whole egg	82.3 ± 3.7	87.9 ± 1.1
	Albumen	75.7 ± 2.0	89.1 ± 1.1
	Yolk	76.6 ± 2.2	84.6 ± 2.5
	Pigeon whole egg	83.2 ± 3.6	86.2 ± 2.2
	Quail whole egg	79.3 ± 1.2	86.4 ± 1.5

**Table 3:** Linearity, determination coefficient, limit of detection (LOD) and limit of quantification (LOQ) of spectinomycin and lincomycin in poultry eggs.

Analyte	Matrix	Regression Equation	Determination Coefficient (*R*^2^)	Linear Range (μg/kg)	LOD (μg/kg)	LOQ (μg/kg)
Spectinomycin	Hen whole egg	*y* = 1251*x* + 16346	0.9993	6.0–2000	3.1	6.0
	Albumen	*y* = 1298.4*x* + 34049	0.9992	6.4–2000	3.0	6.4
	Yolk	*y* = 1199.1*x* − 4540.7	0.9997	5.6–2000	2.3	5.6
	Duck whole egg	*y* = 1059.5*x* − 3709.3	0.9999	6.3–2000	3.5	6.3
	Albumen	*y* = 1113.1*x* − 4463.4	0.9994	7.9–2000	3.8	7.9
	Yolk	*y* = 1051.5*x* − 2573.8	0.9993	8.0–2000	2.7	8.0
	Goose whole egg	*y* = 1113.5*x* − 3245.8	0.9996	7.1–2000	3.5	7.1
	Albumen	*y* = 1179.6*x* − 3611.7	0.9995	7.8–2000	3.2	7.8
	Yolk	*y* = 1074.7*x* − 2777.5	0.9996	6.7–2000	3.0	6.7
	Pigeon whole egg	*y* = 1022.9*x* − 6310.6	0.9991	8.0–2000	4.0	8.0
	Quail whole egg	*y* = 1073.1*x* − 4316.6	0.9998	7.6–2000	3.8	7.6
Lincomycin	Hen whole egg	*y* = 10074*x* − 29019	0.9994	8.4–200	3.1	8.4
	Albumen	*y* = 7829.4*x* + 98669	0.9992	6.7–200	2.6	6.7
	Yolk	*y* = 13453*x* − 27487	0.9994	5.9–200	2.5	5.9
	Duck whole egg	*y* = 8127.3*x* − 16359	0.9996	6.5–200	2.8	6.5
	Albumen	*y* = 7772.3*x* − 312.45	0.9992	7.3–200	3.0	7.3
	Yolk	*y* = 7759.2*x* + 32016	0.9992	8.0–200	4.3	8.0
	Goose whole egg	*y* = 8825.4*x* + 2625.7	0.9996	8.5–200	3.5	8.5
	Albumen	*y* = 9240.1*x* + 6253.1	0.9994	9.0–200	3.8	9.0
	Yolk	*y* = 8381.4*x* + 27696	0.9994	9.2–200	4.0	9.2
	Pigeon whole egg	*y* = 8578.4*x* − 37597	0.9993	9.5–200	3.9	9.5
	Quail whole egg	*y* = 8736.2*x* − 61525	0.9994	8.2–200	4.3	8.2

**Table 4:** Recovery and precision of spectinomycin and lincomycin spiked in blank poultry eggs (whole egg, *n* = 6).

Analyte	Matrix	Spike Level (μg/kg)	Recovery (%)	RSD (%)	Intraday RSD (%)	Interday RSD (%)
Spectinomycin	Hen whole egg	6.0	82.9 ± 1.5	1.8	3.0	4.2
		1000.0	84.5 ± 1.0	1.2	4.1	3.6
		2000.0 ^a^	86.0 ± 1.5	1.7	4.0	4.1
		4000.0	95.7 ± 1.2	1.3	3.7	5.5
	Duck whole egg	6.3	80.9 ± 1.3	1.6	4.6	5.0
		1000.0	85.5 ± 1.5	1.8	3.6	4.5
		2000.0 ^a^	86.1 ± 2.7	3.1	2.9	3.2
		4000.0	89.4 ± 1.8	2.0	2.9	3.4
	Goose whole egg	7.1	82.1 ± 1.7	2.1	3.2	5.0
		1000.0	86.8 ± 1.3	1.5	2.7	4.9
		2000.0 ^a^	87.7 ± 1.7	1.9	2.5	3.0
		4000.0	90.6 ± 1.5	1.7	4.0	4.1
	Pigeon whole egg	8.0	82.3 ± 1.3	1.6	3.3	6.7
		1000.0	83.5 ± 1.1	1.3	3.1	4.6
		2000.0 ^a^	87.7 ± 2.2	2.5	3.7	4.8
		4000.0	88.7 ± 1.3	1.5	2.5	3.7
	Quail whole egg	7.6	85.1 ± 1.4	1.6	3.1	6.0
		1000.0	85.3 ± 1.6	1.9	3.2	4.4
		2000.0 ^a^	87.1 ± 1.9	2.2	3.0	3.9
		4000.0	94.8 ± 1.1	1.2	2.5	3.5
Lincomycin	Hen whole egg	8.4	82.7 ± 1.3	1.6	4.6	5.7
		25.0	85.4 ± 2.4	2.8	5.0	5.6
		50.0 ^a^	84.8 ± 1.6	1.9	3.1	4.5
		100.0	87.1 ± 1.9	2.2	3.2	4.9
	Duck whole egg	6.5	81.8 ± 1.1	1.3	4.3	4.7
		25.0	86.7 ± 2.5	2.9	3.7	5.4
		50.0 ^a^	85.4 ± 2.6	3.0	2.7	3.7
		100.0	86.1 ± 2.7	3.1	4.6	5.0
	Goose whole egg	8.5	80.1 ± 1.5	1.9	2.2	4.1
		25.0	84.0 ± 2.6	3.1	3.4	4.1
		50.0 ^a^	87.9 ± 1.1	1.3	2.3	3.7
		100.0	93.5 ± 1.8	1.9	3.5	3.9
	Pigeon whole egg	9.5	81.4 ± 1.6	2.0	3.9	6.5
		25.0	82.2 ± 2.1	2.6	2.3	3.1
		50.0 ^a^	86.2 ± 2.2	2.6	5.6	6.7
		100.0	91.3 ± 1.6	1.8	3.0	3.9
	Quail whole egg	8.2	80.0 ± 1.3	1.6	2.6	3.3
		25.0	86.7 ± 2.1	2.4	3.9	3.7
		50.0 ^a^	86.4 ± 1.5	1.7	2.6	4.4
		100.0	87.3 ± 1.1	1.3	3.5	5.5

Note: ^a^ Maximum residue limit (MRL). RSD, relative standard deviation.

**Table 5:** Recovery and precision for spectinomycin and lincomycin spiked in blank poultry eggs (egg albumen and yolk, *n* = 6).

Analyte	Matrix	Spike Level (μg/kg)	Recovery (%)	RSD (%)	Intraday RSD (%)	Interday RSD (%)
Spectinomycin	Hen egg albumen	6.4	83.8 ± 2.4	2.9	3.0	4.6
		1000.0	84.2 ± 2.3	2.7	2.9	3.7
		2000.0 ^a^	86.6 ± 2.0	2.3	2.8	3.6
		4000.0	93.0 ± 1.9	2.0	2.2	4.3
	Yolk	5.6	83.9 ± 2.5	3.0	3.5	3.7
		1000.0	84.1 ± 1.7	2.0	3.0	3.4
		2000.0 ^a^	86.6 ± 2.1	2.4	3.7	5.5
		4000.0	94.5 ± 1.4	1.5	2.6	3.8
	Duck egg albumen	7.9	83.6 ± 1.3	1.6	2.7	4.7
		1000.0	84.7 ± 1.5	1.8	2.0	2.2
		2000.0 ^a^	85.7 ± 2.2	2.6	3.0	3.9
		4000.0	87.3 ± 3.0	3.4	5.2	6.5
	Yolk	8.0	82.9 ± 1.9	2.3	2.8	3.5
		1000.0	85.3 ± 2.1	2.5	3.9	4.2
		2000.0 ^a^	87.1 ± 1.3	1.5	2.8	3.3
		4000.0	90.3 ± 2.4	2.7	4.3	6.2
	Goose egg albumen	7.8	80.4 ± 1.5	1.9	2.0	3.7
		1000.0	84.0 ± 2.5	3.0	3.3	6.3
		2000.0 ^a^	83.4 ± 1.4	1.7	2.5	3.1
		4000.0	88.1 ± 1.8	2.0	3.9	4.1
	Yolk	6.7	81.6 ± 2.4	2.9	3.1	3.4
		1000.0	82.4 ± 2.0	2.4	2.4	3.2
		2000.0 ^a^	85.9 ± 1.2	1.4	2.0	3.5
		4000.0	89.4 ± 1.9	2.1	3.1	4.9
Lincomycin	Hen egg albumen	6.7	82.2 ± 1.2	1.5	2.8	3.4
		25.0	85.2 ± 1.6	1.9	3.2	4.1
		50.0 ^a^	85.8 ± 2.2	2.6	3.4	4.5
		100.0	86.5 ± 2.5	2.9	3.5	5.7
	Yolk	5.9	82.7 ± 1.4	1.7	3.9	4.8
		25.0	85.4 ± 2.0	2.3	3.0	3.6
		50.0 ^a^	84.8 ± 2.0	2.4	2.6	3.8
		100.0	91.2 ± 2.1	2.3	4.1	5.5
	Duck egg albumen	7.3	84.9 ± 1.5	1.8	1.9	3.5
		25.0	91.2 ± 2.4	2.6	6.0	5.5
		50.0 ^a^	93.4 ± 2.3	2.5	3.7	4.3
		100.0	95.1 ± 2.2	2.3	3.2	4.9
	Yolk	8.0	85.2 ± 1.3	1.5	3.2	5.3
		25.0	86.5 ± 0.9	1.0	2.5	3.6
		50.0 ^a^	87.9 ± 2.1	2.4	3.8	4.1
		100.0	89.1 ± 2.3	2.6	3.5	3.7
	Goose egg albumen	9.0	80.8 ± 1.6	2.0	2.8	5.3
		25.0	85.4 ± 1.2	1.4	2.6	4.2
		50.0 ^a^	89.1 ± 1.1	1.2	2.7	3.5
		100.0	92.1 ± 1.2	1.3	3.9	3.6
	Yolk	9.2	80.2 ± 1.9	2.4	4.8	4.4
		25.0	85.6 ± 2.1	2.5	4.3	3.9
		50.0 ^a^	84.6 ± 2.5	3.0	4.0	4.1
		100.0	87.9 ± 1.6	1.8	2.7	4.1

Note: ^a^ MRL.

**Table 6:** Comparison of the present method with other methods.

Detection Method	Sample Preparation Method	Analyte	Animal-Derived Food	Analysis Time (min)	LOD (μg/kg)	LOQ (μg/kg)	Recovery (%)
HPLC-FLD [16]	SPE	Spectinomycin	Swine, calf and chicken plasma	12.0	-	-	91.0–104
HPLC-UVD [18]	CSMISPE	Lincomycin	Milk	9.0	20.0	80.0	80.0–89.0
HILIC-MS/MS [22]	LLE	Spectinomycin	Feedstuffs	10.0	-	-	80.0–92.0
HPLC-MS [23]	SPE	Lincomycin	Honey	10.0	7.0	10.0	102–105
HPLC-MS/MS [24]	LLE	Lincomycin	Animal tissues and milk	10.0	1.5–8.8	25.0–50.0	93.9–107
HPLC-MS/MS [27]	ASE	Lincomycin	Meat and milk	40.0	5.0–10.0	10.0–15.0	86.0–91.0
GC-NPD [30]	ASE	Spectinomycin Lincomycin	Animal tissues	13.0	8.1–9.4	16.4–21.4	73.0–97.0
GC-MS [30]	ASE	Spectinomycin Lincomycin	Animal tissues	15.0	1.9–3.1	4.7–5.7	70.0–93.0
GC-MS/MS	ASE	Spectinomycin Lincomycin	Poultry eggs	10.8	2.3–4.3	5.6–9.5	80.0–95.7

Note: “-” Not reported. FLD, fluorescence detection; UVD, ultraviolet detection; NPD, nitrogen phosphorus detection; CSMISPE, core-shell molecularly imprinted solid-phase extraction.
